# Supplementary material for: Bulk RNA-seq deconvolution heterogeneity across paired pancreatic cancer human samples
Source: Front Genet. 2025 Dec 1;16:1662924. doi: 10.3389/fgene.2025.1662924 (PMC12702501; doi:10.3389/fgene.2025.1662924)
Supplement: Supplementary file 1 [file DataSheet1.pdf]

Supplemental Files

Figure S1a. Comparisons of “normalized”, “batch corrected (no normalization)”, “batch and normalized”, and “batch and log2 normalized” gene expression of Mayo (left) and TCGA (right) paired samples for four PDAC specific genes.

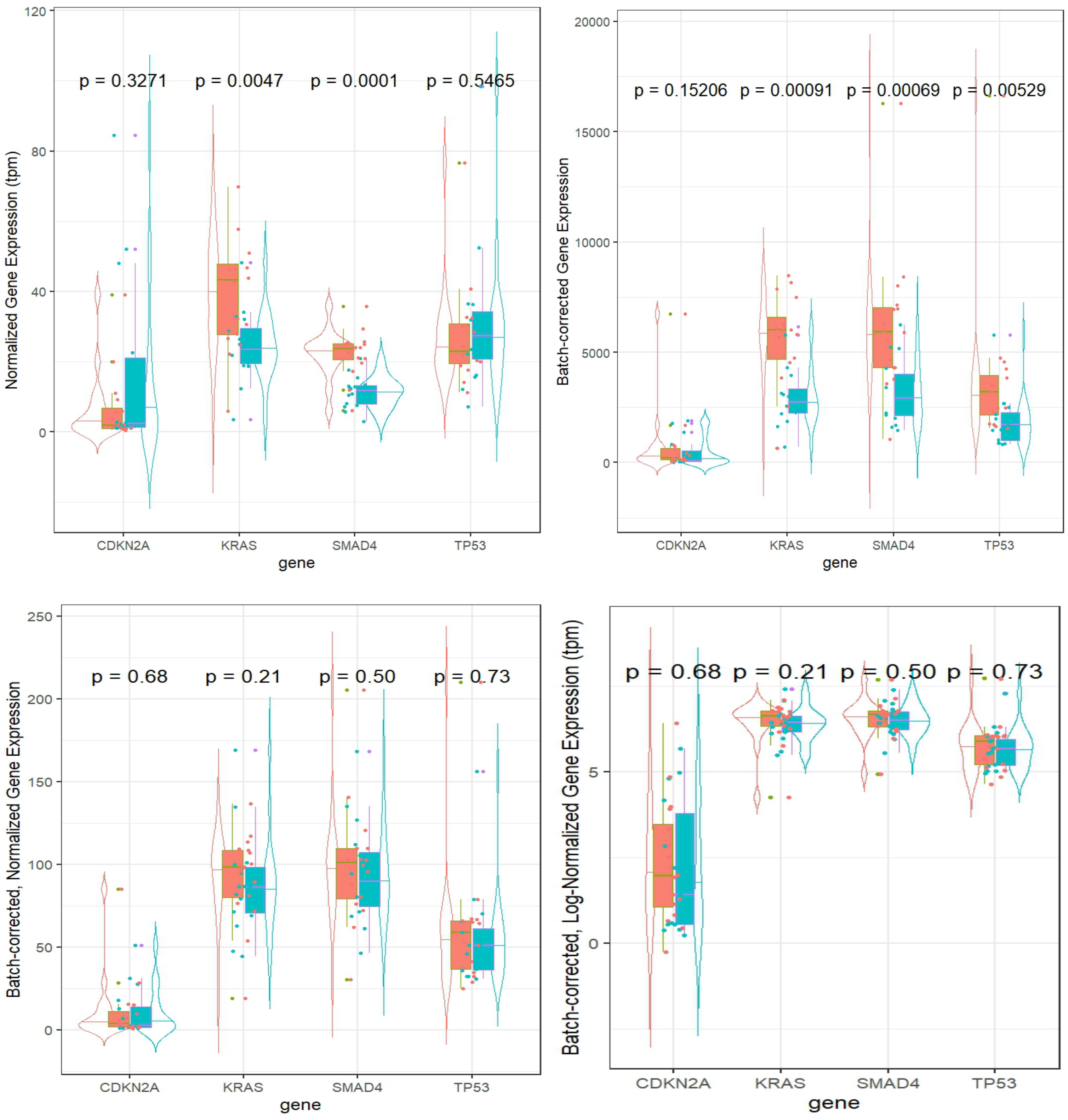

Figure S1b. Comparisons of "normalized", "batch corrected (no normalization)", "batch and normalized", "batch and log2 normalized" gene expression of Mayo (left) and TCGA (right) paired samples for five genes commonly mutated in PDAC.

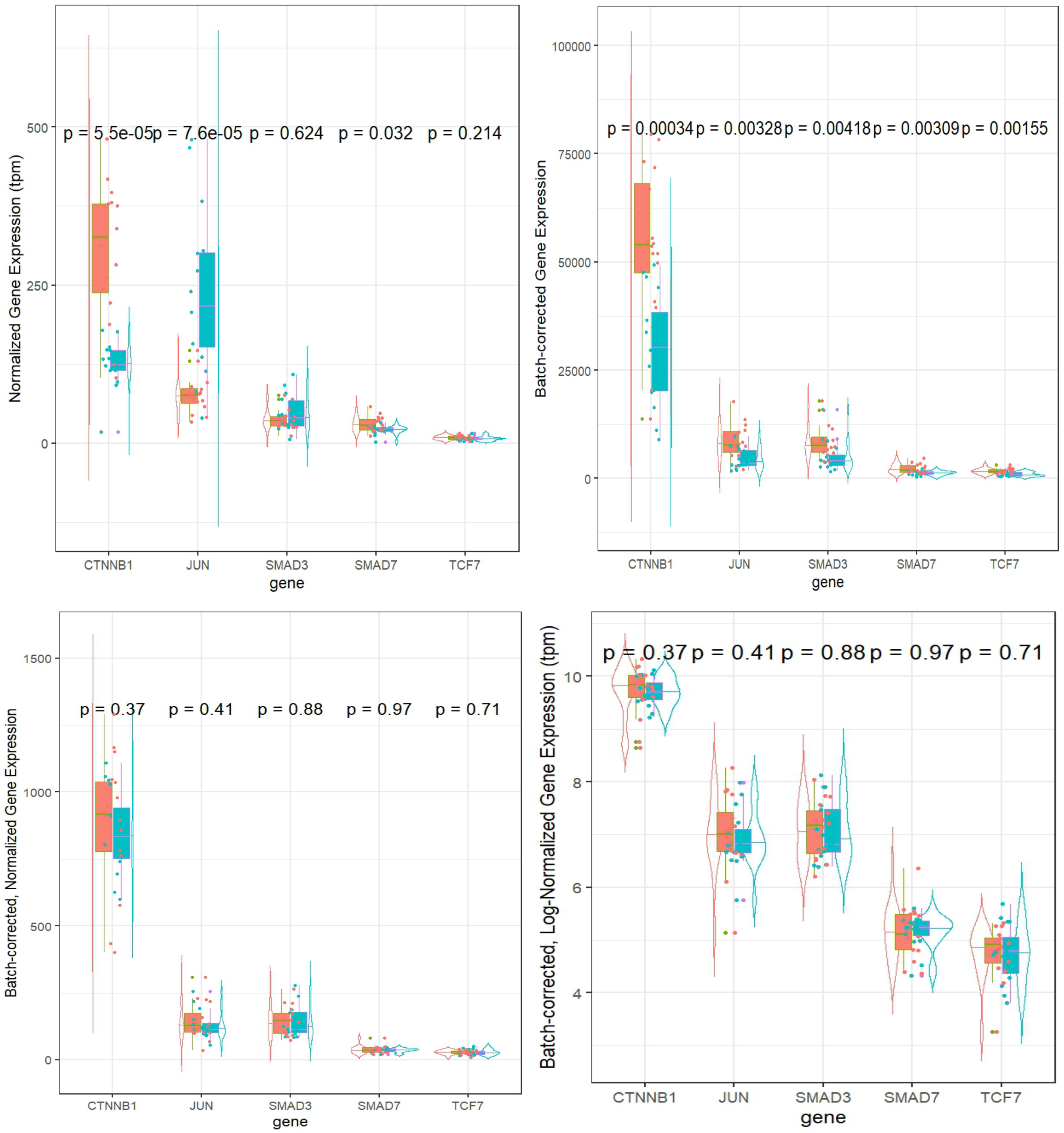

Table S1. Kappa statistic and p-value across selected genes

Gene	Kappa	p-value
KRAS	0.5	0.0455
TCF7	0.25	0.317
SMAD7	0.25	0.317
TP53	0.5	0.0455
SMAD4	0.25	0.317
SMAD3	0.5	0.0455
CTNNB1	0.25	0.317
JUN	0.75	0.0027
CDKN2A	0. 5	0.0455
